# Antibody avidity to pertussis toxin after acellular pertussis vaccination and infection

**DOI:** 10.1080/22221751.2023.2174782

**Published:** 2023-02-16

**Authors:** Aapo Knuutila, Tine Dalby, Niina Ahvenainen, Alex-Mikael Barkoff, Charlotte Sværke Jørgensen, Kurt Fuursted, Jussi Mertsola, Qiushui He

**Affiliations:** a Institute of Biomedicine, University of Turku, Turku, Finland; b Statens Serum Institut, Copenhagen, Denmark; c Department of Pediatrics and Adolescent Medicine, Turku University Hospital, Turku, Finland; d InFLAMES Research Flagship Center, University of Turku, Turku, Finland

**Keywords:** Pertussis, pertussis toxin, avidity, ELISA, vaccination

## Abstract

Pertussis toxin (PT) is a unique virulence factor of *Bordetella pertussis*, and therefore a key component of acellular pertussis vaccines. Although immunity after infection seems to persist longer than after vaccination, the exact mechanisms are not fully known. In this study the overall binding strength (avidity) of anti-PT IgG antibodies was compared after acellular booster vaccination and infection, as a parameter to evaluate long-lasting protection.

Danish and Finnish serum samples from a total of 134 serologically confirmed patients and 112 children who received acellular booster vaccines were included in this study. The concentration of anti-PT IgG was first determined by ELISA, followed by two separate ELISAs to evaluate antibody avidity: either with a dilution series of urea as a bond-breaking agent of antibody and antigen binding and a constant anti-PT IgG concentration between the samples or with a constant dilution ratio of sera and detergent. In addition to urea, the use of diethylamine and ammonium thiocyanate as disruptive agents were first compared between each other.

A strong Spearman correlation (*R* > 0.801) was noted between avidity and concentration of anti-PT IgG antibodies if a constant serum dilution method was used, and avidity was noted to be higher in patients in comparison to vaccinees in Denmark, but not in Finland. However, no correlation between antibody concentration and avidity was found if a constant anti-PT IgG concentration was used (*R* = −0.157). With this method, avidity after vaccination was significantly higher in comparison to that after infection in both Danish and Finnish subjects (*p* < 0.01). A shorter time since the latest booster vaccination was found to affect avidity positively on the next PT-antigen exposure with either vaccination or infection.

## Introduction

Primary and booster immunizations with acellular pertussis vaccines (aP), containing pertussis toxin (PT), pertactin, filamentous hemagglutinin, and fimbriae, are commonly used for protection against pertussis disease by the bacterium *Bordetella pertussis*. Despite a high vaccination coverage, *B. pertussis* still causes disease among children and adults [1,2], and worrisomely, pertussis incidence has increased in recent years, particularly in older children, adolescents, and adults [2–5]. The lack of defined and appropriate immune correlates for protection has been a challenge when evaluating the protective efficacy of the next-generation aPs [6,7]. Thus far, studies with aPs illustrate that serological correlates against pertussis toxin, pertactin, and fimbrial antigens contribute to the protection against pertussis infection [7,8]. However, the different functions of antibodies such as neutralization of bacterial antigens or prevention of bacterial binding to epithelial cells may still be inferior after vaccination in comparison to after natural infection [6]. Although antibody concentrations decline rapidly over time after immunization, the duration of humoral immunity after vaccination seems at least as long as after infection at 2–6 years [9,10], whereas cell-mediated immunity is maintained for several years [11–13].

After antigen exposure, either by vaccination or infection, the humoral immune response to a specific antigen consists of the maturation of the quantity and quality of antibodies over time [14,15]. In terms of quality, parameters such as affinity/avidity mainly measure the binding strength of antigen-specific antibodies which thereby determine the efficiency of the circulating antibodies [16]. This may act as a correlate for long-term immunity [17,18] and protection against pertussis [19]. Avidity maturation evolves as an antigen-driven selection process [20,21] within the germinal centres [22,23], and as a result of somatic hypermutation [24] leads to the progressive selection of high-affinity antibodies [25–28]. Thus, avidity maturation may serve as a surrogate marker for memory priming and clonal selection of high-affinity memory B cells [25,26], and a high avidity would potentially indicate individuals who are primed for long-lasting memory [25].

Avidity index (AI) is commonly used to characterize the functionality of antibodies based on the proportion of strong binding of antibodies to an antigen after treating the antibodies in chaotropic solutions. Generally, by ELISAs, a constant dilution or a titrated sample is incubated, and the formed complexes are exposed to single concentrations of the chaotropic agent. Alternatively, a fixed concentration of antibodies is incubated with increasing concentrations of the chaotropic agent [29,30]. In the field of pertussis, most studies have focused on investigating avidity from vaccination measured in anti-PT IgG antibodies. These studies claim that avidity towards PT increases after vaccination [15,19,27], which is affected by the previous priming by either aP or whole-cell vaccines [31], and that avidity declines alongside overall anti-PT IgG concentration over time since primary vaccination [32]. In regards to infection, a study by Barkoff et al. demonstrated that similar to aP boosting, a sharp increase in avidity can be observed between the first and second sera of culture-confirmed patients, and a study by Hovingh et al. demonstrated that the attained avidity remains high after the symptomatic phase up to years after recovery [19,33]. Yet a principal question remains whether vaccination-induced antibodies are as good as those from infection regarding avidity. And if they are not, does this hamper the full capability of vaccines to protect against the disease?

Hence, a thorough comparison of antibody avidity between different exposure backgrounds may aid in the development of future vaccines as more precise correlates of protection. It has been suggested that studies of vaccine efficacy should incorporate analyses of avidity [34,35]. However, in the literature, there is large heterogeneity in the experimental methods for the determination of avidity, thereby raising questions on the most appropriate procedures. Furthermore, a practical limitation on the used methodology may occur when assessing samples with high total antibody concentrations, as significant decreases in antibody binding cannot be seen reliably [29].

**Figure 1.**
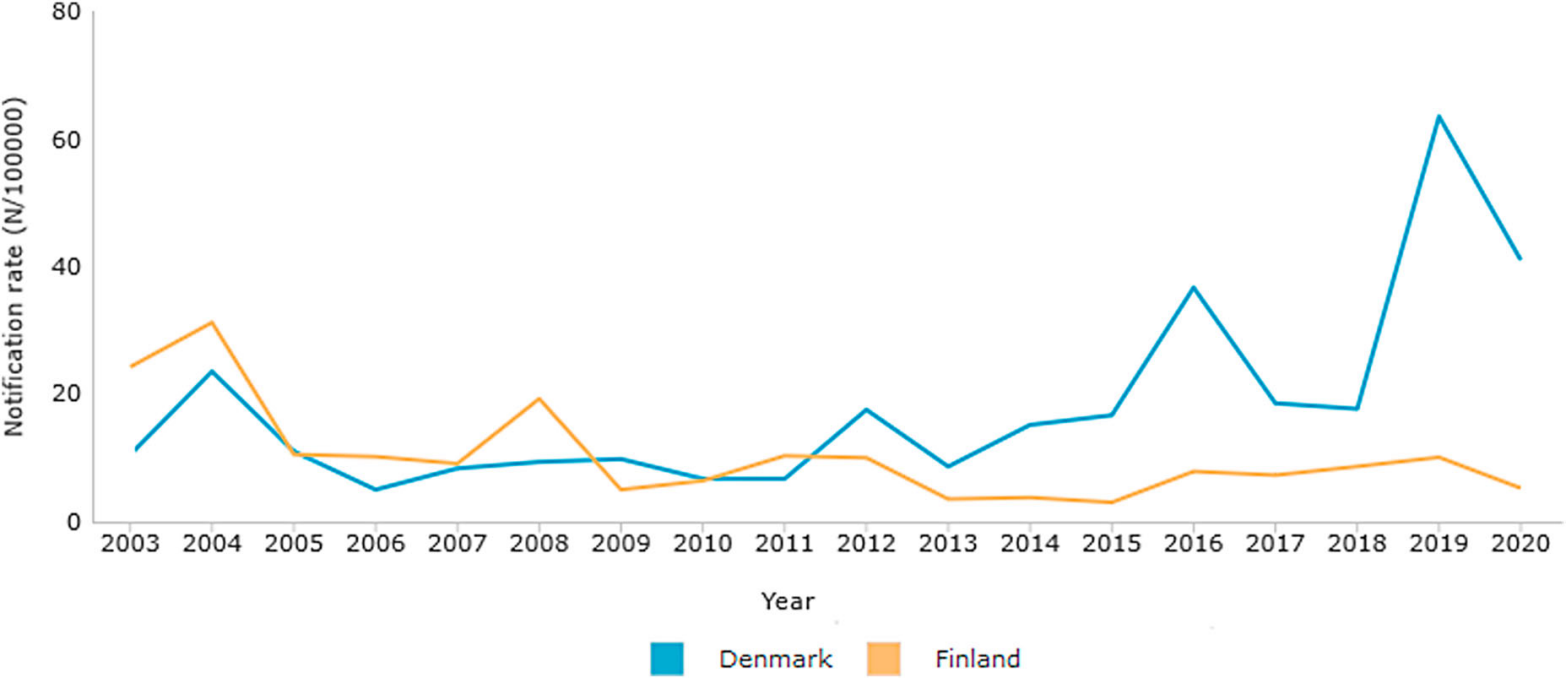
Laboratory confirmed notification rates of pertussis in Denmark (Blue) and Finland (Orange) during the acellular vaccination era.

In Finland, aP vaccine containing a glutaraldehyde-detoxified PT (Pentavac®, Sanofi Pasteur MSD, France) was used from 2005 to 2008, and Infanrix® (GlaxoSmithKline, Belgium) containing formaldehyde-detoxified PT was used from 2009 to 2019 for primary immunizations. In Denmark, a vaccine containing only hydrogen peroxide-detoxified PT was used since 1997 until 2019 (DiTeKiPol or DiTeKiPol/Act-Hib, previously Statens Serum Institut, now AJ Vaccines). In both countries, children receive a booster dose at 4–5 years of age, and in Finland, an additional booster is given at 14 years of age. During the acellular vaccine era, the number of pertussis cases has also varied between the two countries as shown in Figure 1.

We aimed to specifically study if overall anti-PT IgG titers affect avidity with two methodological approaches, and to show whether avidity varies between infected and vaccinated individuals in two countries with different vaccines and pertussis incidence history.

## Materials and methods

### Study samples

Table 1 chosen for this study were selected to have at least 25 IU mL^−1^ of anti-PT IgG-antibodies. Five different categories of sera were analyzed, three from Denmark and two from Finland. The study included sera from Danish adolescents with confirmed pertussis by serology (DK-P) [36], who were vaccinated in childhood with the Danish PT-only aP vaccines (DiTeKiPol/Act-Hib and DiTeKiPol, Statens Serum Institut / AJ Vaccines) at the schedule 3m + 5m + 12m + 5y, sera from Danish children, who had recently received their third primary dose of PT-only aP vaccine (DiTeKiPol/Act-Hib) at the age of 1 (DK-1), and sera from Danish children who had recently received a booster dose of the PT-only vaccine (DiTeKiPol) at the age of 5 years (DK-5). From Finland, sera from serologically diagnosed pertussis patients (from the period 2015–2016, based on IgA and IgM antibody concentrations to sonicated *B. pertussis* bacteria [37] in combination with anti-PT IgG antibody concentrations) (F-P), and sera from Finnish children from a vaccination cohort (“BERT”) who had received a booster dose of Tdap3-IPV vaccine (BoostrixTM-IPV – GlaxoSmithKline (GSK), Wavre, Belgium), and whose sera were collected one month after vaccination (F-8/15) [38]. All sera in this study were stored either at −20 or −70°C and their anti-PT IgG antibodies were measured with standardized ELISA at the Finnish National Reference Laboratory for Pertussis as previously described [19,39].

**Table 1.** Study subjects.

Group	*N*	Age, years (median, range)	Sex (F/M)	Anti-PT IgG, IU mL^−1^ (median, range)	Days since last vaccination (median, range)
Danish children, primary vaccine (DK-1)	20	1.5 (1.1–2.1)	7/13	47 (25–252)	158 (15–413)
Danish children, booster vaccine (DK-5)	34	5.7 (5.0–6.9)	18/16	113 (30–559)	174 (10–677)
Danish patients (DK-P)	39	13.5 (10.2–18.9)	21/18	257 (43–769)	3262 (5–13 years)
Finnish children, booster vaccine* (F-8/15)	58	11.1 (8.5–15.8)	29/29	226 (25–1432)	28 (26–30)
Finnish patients (F-P)	95	13.5 (3–66.5)	13/13**	202 (26–1041)	not available

* From a clinical pertussis booster study of BERT conducted in Finland [38].

** Data available from 26 participants.

### Study approval

The regional research ethics committee in Denmark did not require discrete approval for the use of Danish sera for this study. The BERT clinical study was registered at the European Clinical Trials register under the study number: 2016-003678-42 and approved by the medical research ethics committee of Turku University Hospital (ETMK Dnro: 129/1800/2017). Parents or legal guardians of minor participants provided written informed consent. The study was designed and conducted in compliance with the principles of the Declaration of Helsinki (1996). For the use of Finnish patient samples, permission was granted by the Ethics Committee of the Hospital District of Southwest Finland by the chief of the operative group of Turku University Hospital (Decision 14/17 MBG).

### Avidity assay optimization

**Figure 2.**
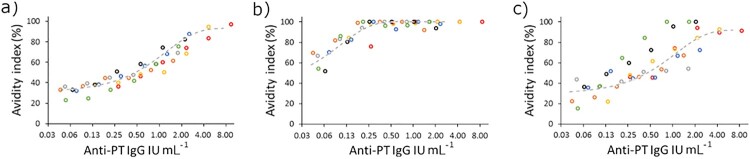
Seven samples (marked with individual colours) with varying anti-PT IgG concentrations were tested in a dilution series with different detergents for avidity: (a) 6.5 M urea, (b) 1 M ammonium thiocyanate and (c) 30 mM DEA. The nonlinear curve fit (dashed grey line) of the samples was plotted with Origin, V. 2016 (OriginLab Corporation, Northampton, MA, USA.)

The concentration of antibodies in the sample, as well as of detergent, was addressed for optimization. The assays were performed as described in the next chapter: “Avidity assays.” A strong correlation was noted between avidity and antibody concentrations when seven serum samples, selected from Finnish patients and vaccinees, with varying initial anti-PT IgG concentrations were diluted in series with urea, diethylamine (DEA), and ammonium thiocyanate (Figure 2). Based on the dilution series, avidity remained unchanged in the samples at a limit below 0.25 IU mL^−1^ corresponding to 0.025 anti-PT IgG IU per well with urea and DEA, but with ammonium thiocyanate, such a limit could not be defined with the used dilution series. The serum concentration of 0.025 IU per well was selected for the following experiments since smaller amounts might occasionally result in too diluted samples, which in return led to very low differences between detergent and PBS wells, resulting in high avidity values. Urea was selected as the detergent for the final assay based on good linearity and replication.

### Avidity assays

The avidity of serum antibodies was determined by ELISA [19]: First, 96-well plates (Greiner Microlon, art. No. 655061, Frickenhausen, Germany) were coated overnight with 200 ng of purified native PT (GlaxoSmithKline, Belgium) in 100 µL of PBS (pH 7.4). After the wells were washed once with 0.9% NaCl-0.05% Tween (Sigma P-1379, St. Louis, USA) washing buffer, the plates were blocked with 1% BSA-PBS (cat no 810033; MP Biomedicals, Solon, Ohio, USA) for one hour at 37°C. Next, after three washes, the serum samples were diluted either by a constant 1:50 dilution or to match a fixed concentration of 0.025 IU per well of anti-PT IgG in 100 µL of 1% BSA-PBS, the diluted sera were then added in duplicate wells and incubated for two hours at 37°C. Blank samples (1% BSA-PBS and PBS) and anti-PT IgG negative and positive controls from in-house serum sample pools with known concentrations of anti-PT IgG antibodies were included on each test plate and were used to adjust for inter-assay variation. After washing the plates thrice, half of the wells were treated with 100 µl of either 2–8 M urea, 30 mM DEA or 1 M ammonium thiocyanate in a constant or a dilution series of a detergent, and the other half with PBS for 15 min. Similar ranges of detergents are widely used in the literature [19,27,29,31–33,40–45]. Wells were washed thrice, and goat anti-human IgG conjugate (AP124, Merck, Espoo, Finland) was then diluted 1:2000 in 100 µl of BSA-PBS and incubated for one hour at 37°C. Last, after three washes, 100 µL of p-Nitrophenylphosphatase substrate (cat no S0942, Sigma, Helsinki, Finland) in diethanolamine-MgCl_2_-buffer (Reagena, cat no 170057, Toivala, Finland) was incubated and covered from light for six minutes before the reaction was stopped with 100 µl of 3 M NaOH. Absorbance was measured at 405 nm with Victor X4 device (PerkinElmer, Turku, Finland). The avidity index was defined as the ratio of absorbance of the sample treated in detergent divided by the absorbance of the sample in a PBS well from background reduced signals.

### Statistics

AI values were analyzed using IBM SPSS statistics 27.0 software for Windows (IBM Corp., Armonk, NY, USA). AI values over 100% were converted to 100.0% for data analysis. The differences in means between the groups were tested with ANOVA or Student’s t-test, and two-sided *p*-values less than 0.05 were considered statistically significant. The correlation of AI to the overall anti-PT IgG IU mL^−1^ concentrations was calculated with the Spearman correlation coefficient.

## Results

### Avidity dependency on overall antibody concentrations

**Figure 3.**
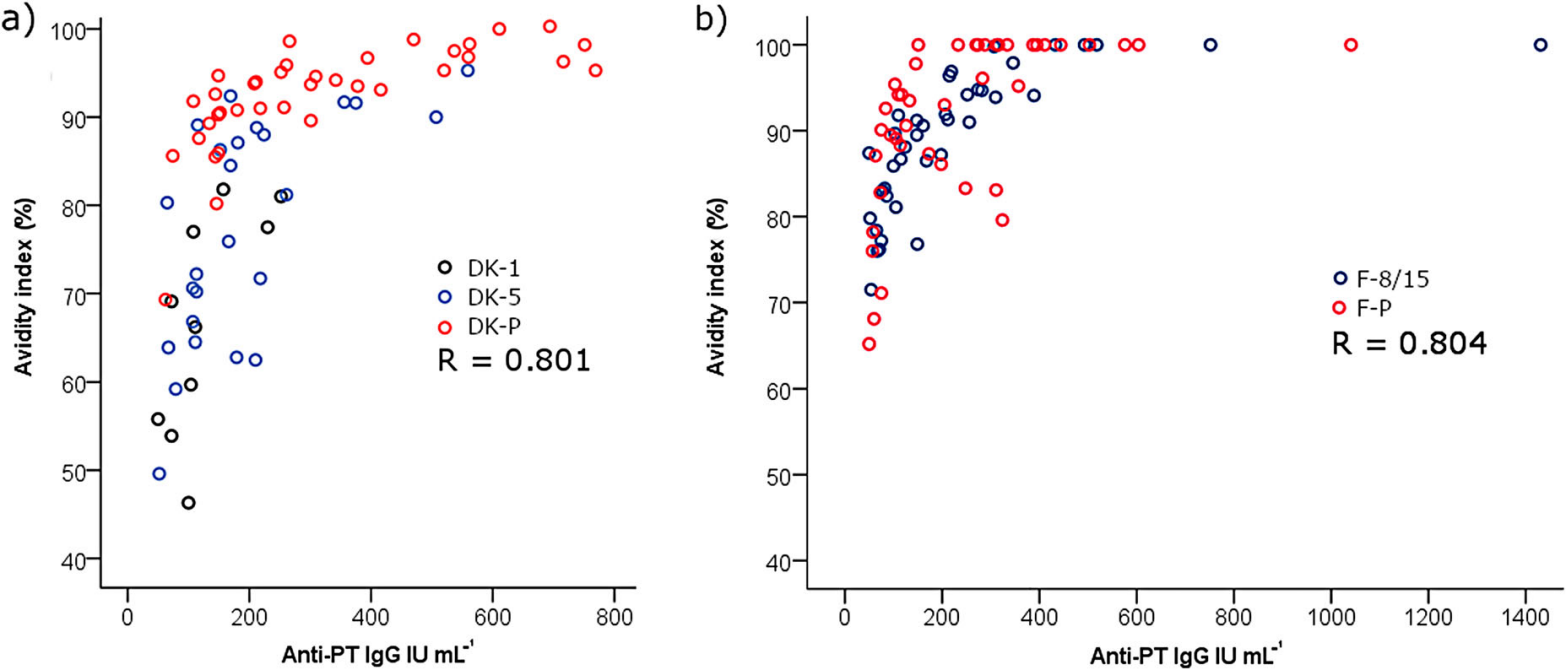
A high correlation between anti-PT IgG and avidity, measured with a constant 1:50 dilution of serum together with 30 mM DEA, was observed across samples from (a) recently vaccinated Danish children, age of 1–2 years (DK-1), and 5–6 years (DK-5), as well as pertussis patients (DK-P) (*N* = 10, 25, 38 respectively), and (b) recently vaccinated Finnish children, age of 8–15 years (F-8/15), and pertussis patients (F-P) (*N* = 42 and 42) were tested. Only samples with higher than 50 IU mL^−1^ anti-PT IgG were included in this test and analysis. R represents Spearman correlation.

**Figure 4.**
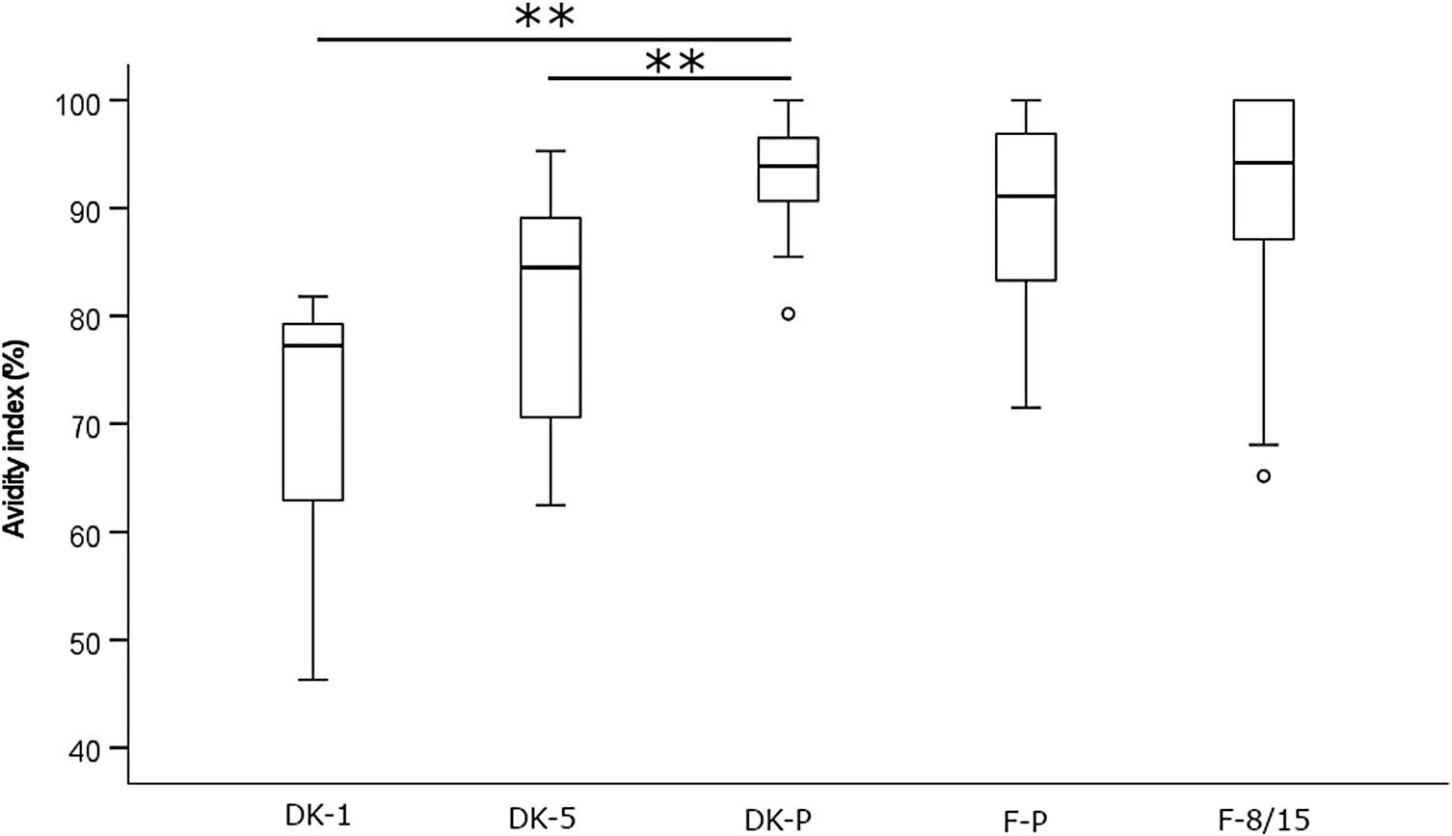
Avidity of anti-PT IgG antibodies was tested by using 30 mM DEA with a constant 1:50 dilution of serum from recently vaccinated Danish children, age of 1–2 years (DK-1), and 5–6 years (DK-5), as well as pertussis patients (DK-P) (*N* = 10, 25, 38 respectively), and recently vaccinated Finnish children, age of 8–15 (F-8/15), and pertussis patients (F-P) (*N* = 42 and 42). Only samples with higher than 50 IU mL^−1^ anti-PT IgG were included in this analysis. Significance by the two-tailed ANOVA for independent samples comparing each study population within countries is indicated as**p < 0.01. The box plots demonstrate the median, quartile range, and 1.5 times the quartile range of inhibition of the study groups. Ο = values exceeding 1.5 times the interquartile range.

The binding strength of anti-PT antibodies induced after infection and acellular vaccination was assessed with ELISA using disrupting detergents as a chaotropic agent. A constant 1:50 dilution ratio of serum samples and DEA as the detergent lead to a high correlation between anti-PT IgG concentrations and avidity (Spearman *R* = 0.801 and 0.804 among the Danish and Finnish study samples, respectively) (Figure 3). The level of avidity (Figure 4) (and overall anti-PT IgG, Table 1) were significantly higher among the Danish patients in comparison to Danish vaccination groups (*p* < 0.01), however, no difference in avidity or anti-PT IgG was noticed between the Finnish groups of patients and vaccinees (Figure 4, Table 1). A similar outcome in avidity was also found with 6.5 M urea instead of DEA with the Finnish samples (*R* = 0.79, data not shown). Due to the noted high correlation between avidity and overall concentrations of anti-PT IgG, these conflicting observations could be due to differences in anti-PT IgG concentrations between the groups (Table 1). If the study cases were separated into two groups based on a threshold of 100 international units per mL (IU mL^−1^) anti-PT IgG, the avidities were significantly higher in each of the >100 IU mL^−1^ groups.

### Avidity assays with a fixed antibody concentration and dilution series of detergent

**Figure 5.**
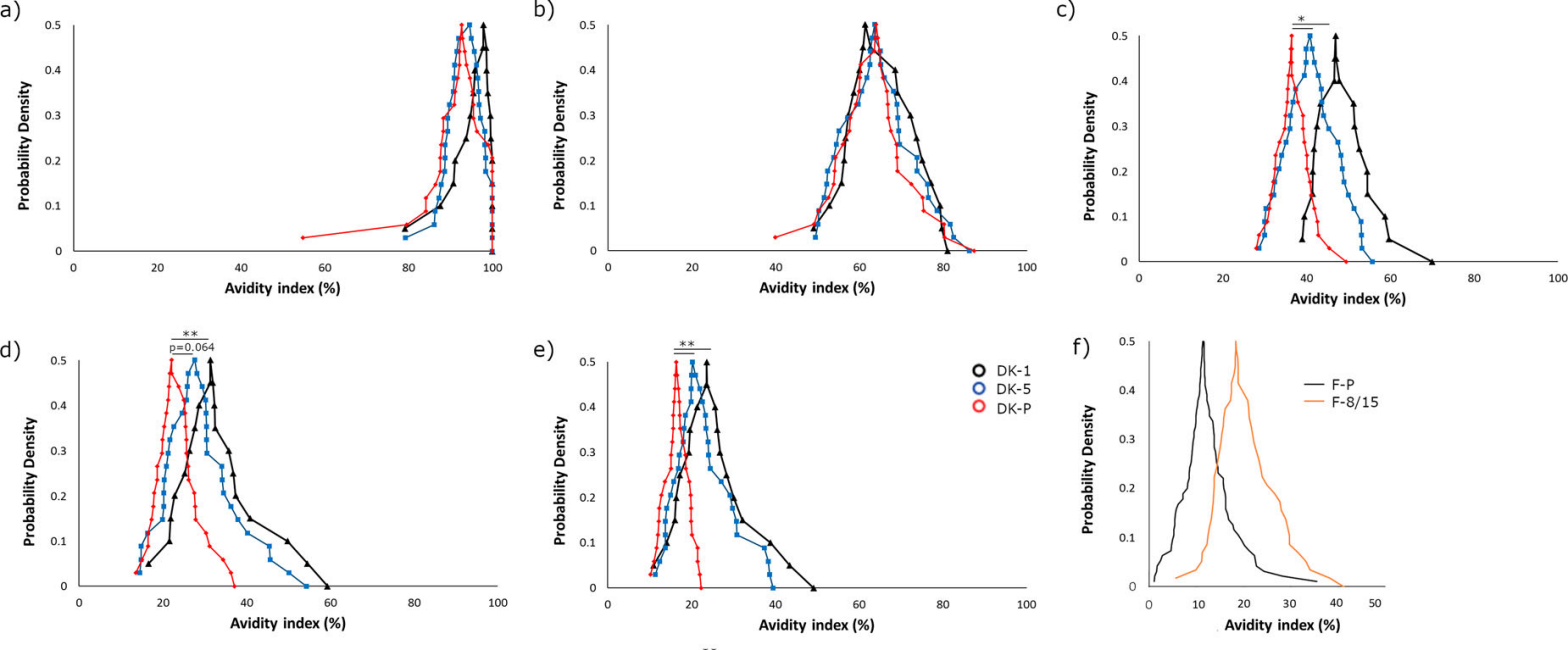
The cumulative distribution of avidity index values of the recently vaccinated Danish children, age of 1–2 (DK-1, black line), and 5–6 (DK-5, blue line) as well as pertussis patients (DK-P, red line) (N = 20, 34, 39 respectively), (Table 1) across different urea concentrations: a) 2 M b) 3.5 M c) 5 M d) 6.5 M e) 8 M. Finnish children, age of 8-15 (F-8/15), and pertussis patients (F-P) (N = 58 and 95, respectively) (Table 1) were measured with 8 M urea (f). Significance by ANOVA is indicated between the groups as **p-value < 0.01, *p-value < 0.05.

**Figure 6.**
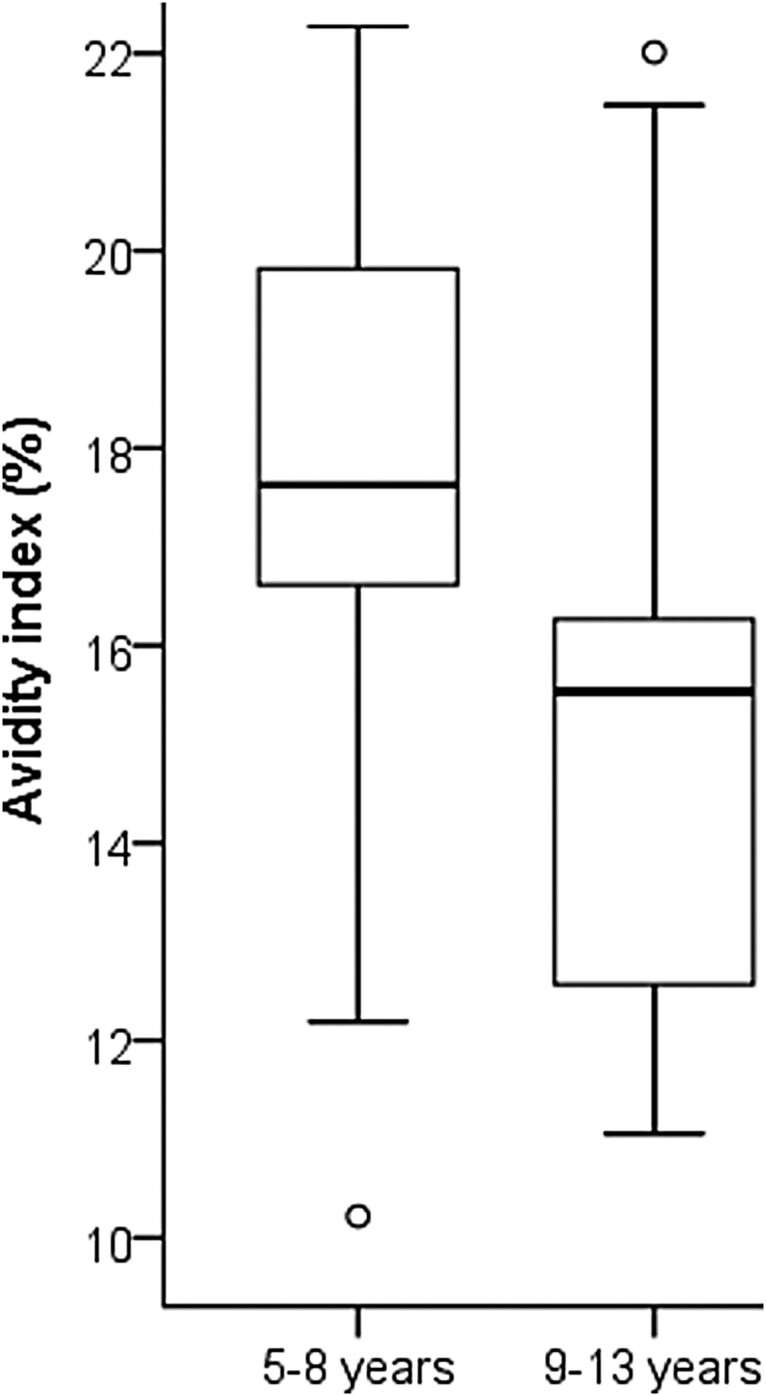
The avidity index of the Danish patients was measured with 8 M urea. Adolescents who had been more recently vaccinated (within 5–8 years, *N* = 20) had significantly higher avidity (t-test, *p* < 0.01) in comparison to vaccination after 9–13 years (*N* = 19). The box plots demonstrate the median, quartile range, and 1.5 times the quartile range of avidity. Ο = values exceeding 1.5 times the interquartile range.

The Danish study samples were tested in the presence of five dilutions of urea ranging between 2 and 8 M (Figure 5). Diluting to a fixed anti-PT IgG concentration resulted in a normal distribution of data for all groups (Shapiro-Wilkins *p* > 0.05). The inter-assay variation was evaluated to be 10.9% using the Danish samples on duplicate runs. There was no correlation between avidity and anti-PT IgG concentrations (*R* = −0.157), and if the signals of the control wells with PBS were considered (which acts as a reference to the overall amount of bound antibodies to PT), there were no differences between the study groups. With lower urea concentrations at 2–3.5 M, there were no differences in avidity-index between the groups, whereas with higher concentrations DK-1 and DK-5 groups had higher avidities in comparison to DK-P (*p* < 0.01) (Figure 5). The DK-1 group had slightly higher avidities in comparison to the DK-5 group. Similar to the Danish study groups, the F-8/15 group had higher avidity with 8M urea than F-P (Figure 5). Within DK-P, those adolescents who had been vaccinated within 5–8 years before the infection had statistically significantly higher avidity across 3.5–8 M urea concentrations in comparison to those adolescents who had been vaccinated 9–13 years before infection (*p* < 0.01) (Figure 6), although they had similar anti-PT IgG concentrations (*p* = 0.710). This difference between vaccination history was not observed with a constant dilution method (*p* = 0.827) since high anti-PT IgG concentrations in this cohort lead to nearly a stagnant >90% avidity response in these participants. A negative correlation was found with the Danish vaccination samples between avidity and the time since the latest vaccination across all urea concentrations (range of Spearman R: [−0.336 to −0.284], *p* < 0.05).

## Discussion

Studies with immunoassay-based methods have shown that anti-PT avidity correlates with antibody concentrations [19,27,41,42]. However, at times, it can be challenging to evaluate avidity due to a very low quantity of antibodies or due to a low difference in antibody concentrations [15,19,32,43]. Problematically, such issues are often left unreported. There are a variety of models to present avidity data ranging from avidity index values, IC50-values (based on either a detergent or a serum dilution series) [29,44], AI thresholds (40–60%) [27], and combinatory models which aim to fractionate respective avidity proportions across detergent ranges or to account for overall antibody IgG titers [46,47]. Since AI is affected by detergent and its concentrations (Figure 5) [29], incubation time, and temperature [15] it is challenging to define arbitrary cutoffs for interpreting high or low antibody avidity [48]. These experimental conditions may further influence the measurements as much as different clinical backgrounds [49], genetics or other environmental factors [38,50]. The variety of data analysis models and assay designs makes it particularly challenging to make consistent conclusions across previous studies. Arguably, the most reliable avidity assays would utilize both dilution series of samples and detergent, to assess these two important parameters for each study population. However, this is reagent, sample, and time-consuming. The approach of detergent dilution series is, at least for now, essential to visualize the whole spectrum of antibodies, as we yet lack the knowledge of a clinically relevant concentration of avidity for protection [6,7,46,47].

Inevitably, initial antibody concentrations and thus sample selection may and will influence the clinically relevant conclusions regarding avidity, which was also demonstrated by the presented data. Hypothetically, the same sample should have the same avidity regardless of antibody amount, as the proportion of strong–weak antibodies is always the same. However, surprisingly, contradictory conclusions on avidity were made solely based on the variation of the initial concentrations of antibodies; based on a constant dilution rate of serum, vaccination-induced antibodies were, at the same time, inferior or equal in avidity in comparison to infection (Figure 4). The high overall amount of antibodies on a capture surface affected the ability of a detergent to interfere with binding, with the surface oversaturating with only strong binding antibodies, roughly with titers higher than 200 IU mL^−1^ (Figure 3), and this concern has also been highlighted by others [15,29]. On the contrary, with a lower concentration of antibodies, detergents seem to interfere more effectively leading to higher individual variation of avidity. Therefore, an approach with a fixed serum concentration [30,51,52] and with a wide range of detergent concentrations was developed in this study. After diluting the sample to the point, where all antigen-specific antibodies with varying affinities within a sample have space to bind on the capture surface, from this immunologic equilibrium, avidity can be reliably evaluated. This was defined as the lowest point of the linear region of the antibody dilution curve (Figure 2). Urea’s ability to intervene with binding was optimal in the range of 5–8 M to separate patient and vaccine backgrounds. Nevertheless, urea at 3.5 M concentration also demonstrated a high range of variation in avidity and may therefore be useful in other study populations. Concentrations below 3.5 M and above 8 M urea were too polarizing to avidity either way, and any differences between groups were lost. It may be possible to constitute more accurate ranges of detergent concentrations (and even single concentrations) to be tested in the future in respective study populations. Nevertheless, a wide range of detergent concentrations is necessary to categorize avidity responses accurately.

With a fixed amount of antibodies between the samples, among both Danish and Finnish samples, the vaccinated children had higher antibody avidity to PT than pertussis patients within a wide range of urea concentrations (Figure 5). Based on previous studies, repeated vaccine doses with a high amount of PT should produce high antibody avidity [15,19,27]. Yet, all patients have very likely received vaccinations in their childhood, and therefore yet another re-exposure to PT should lead to a strong avidity if any immunological memory remains. One explanation lies in the difference of age between the groups, or rather in the timing of the latest vaccination: avidity was the highest in DK-1 (third dose of primary vaccination within 6 months), the second highest within DK-5 (four years from priming), then F-8/15 (4–10 years since latest booster), and finally DK-P with (5–13 years since boosting). A similar age trend was noted by Fumimoto et al [42]. Likewise, within DK-P the highest avidities were in general noted among the younger and thereafter more recently vaccinated subjects. The slight differences between the two countries are possibly reflected upon differences in the vaccines [53]. Although age in general affects the overall capability of antibody maturation [21,24,50,54], our results instead highlight the significance of multiple vaccination doses and the time since vaccination (or reoccurring exposure in general) to avidity, despite lower antibody titers induced by vaccination in young children (Table 1) [45,55,56]. This finding is similar to those observed with anti-PT antibodies [57], anti-PT neutralizing antibodies [58], and PT-related memory B-cells [59], studies which demonstrated that existing memory increased the immune response after vaccination to PT.

The sampling time of vaccinated subjects was well-defined in the study, whereas the timing of serologically diagnosed patients’ sampling may highly vary from weeks to possibly even years after the actual infection. Unfortunately, no data regarding vaccination history were available for most of the Finnish patients to further confirm the effect of the recency of vaccination on avidity response upon infection. Preferably, patient samples with a diagnosis either by PCR or culture would be more suited for future studies, as these will be at a similar stage in the course of the infection. Reassuringly though, the finding by Hovingh et al. [33] (with a fixed serum concentration avidity method) demonstrated that avidity remained unchanged from symptomatic infection to years after recovery. Although the sample selection was restricted in our study to samples with higher than 25 IU mL^−1^ anti-PT IgG, no doubt lower concentrations could be tested However, with samples containing very low antibodies (less than 5 IU mL^−1^), the non-specific binding from the high amount of overall sera to be included may interfere with the assay [15]. Of note, measuring low antibody concentrations does not pose significant relevancy for recent clinical pertussis infections, as the recommended diagnostic cut-off of anti-PT IgG antibodies is 100 IU ml^−1^ [60]. Arguably, we have aimed to reduce the effect of overall anti-PT antibody concentrations on avidity, but still, bias might exist as a straight-dilution assay is not always accurate. Nevertheless, reassuringly the fixed serum concentration led to normally distributed data within all the study groups.

In conclusion, inconsiderately chosen methods for avidity measurements combined with the diversity of initial antibody concentrations can on their own have a very high influence on the outcome and claims of clinically relevant observations regarding antibody avidity. With the fixed serum concentration approach, together with a wide detergent concentration range, the effects of different initial antibody concentrations of samples could be nullified, and thus ease the interpretation and comparisons between future studies. With this approach, recent vaccination was found to produce a higher avidity response in comparison to pertussis infection, demonstrating the capability of acellular vaccines in this regard to produce efficient antibodies. Importantly, this finding does not rule out that infection still induced a lot of antibodies (Table 1) with overall high avidity (Figure 3). A shorter period from the latest previous childhood acellular booster vaccination was noted to positively affect the avidity responses at the next PT-antigen exposure whether via vaccination or infection, which may reflect the effectiveness of vaccination-induced protection over time. This study thus provides evidence to evaluate avidity antibody response also at other vaccine and infection studies. Studies that provide a connection between other antibody characteristics and a sufficient, justified, and long-lasting concentration of avidity regarding protection against the disease are needed for future vaccination development to ensure the quality of the induced antibodies.

## References

[1] Elomaa A, He Q, Minh NN, et al. Pertussis before and after the introduction of acellular pertussis vaccines in Finland. Vaccine. 2009;27(40):5443–5449. doi:10.1016/j.vaccine.2009.07.010. 19628060

[2] Mooi FR. Bordetella pertussis and vaccination: the persistence of a genetically monomorphic pathogen. Infect Genet Evol. 2010;10(1):36–49. doi:10.1016/j.meegid.2009.10.007. 19879977

[3] He Q, Mertsola J. Factors contributing to pertussis resurgence. Future Microbiol. 2008;3(3):329–339. doi:10.2217/17460913.3.3.329. 18505398

[4] Witt MA, Katz PH, Witt DJ. Unexpectedly limited durability of immunity following acellular pertussis vaccination in preadolescents in a North American outbreak. Clin Infect Dis. 2012;54(12):1730–1735. doi:10.1093/cid/cis287. 22423127

[5] de Greeff SC, Mooi FR, Westerhof A, et al. Pertussis disease burden in the household: how to protect young infants. Clin Infect Dis. 2010;50(10):1339–1345. doi:10.1086/652281. 20370464

[6] Plotkin SA. Complex correlates of protection after vaccination. Clin Infect Dis. 2013;56(10):1458–1465. doi:10.1093/cid/cit048. 23386629

[7] Cherry JD. Pertussis: challenges today and for the future. PLoS Pathog. 2013;9(7):e1003418. doi:10.1371/journal.ppat.1003418. 23935481 PMC3723573

[8] Olin P, Hallander HO, Gustafsson L, et al. How to make sense of pertussis immunogenicity data. Clin Infect Dis. 2001;33(Suppl 4):288–S291. doi:10.1086/322564. 11709761

[9] Hallander HO, Ljungman M, Storsaeter J, et al. Kinetics and sensitivity of ELISA IgG pertussis antitoxin after infection and vaccination with Bordetella pertussis in young children. APMIS. 2009;117(11):797–807. doi:10.1111/j.1600-0463.2009.02530.x. 19845530

[10] Dalby T, Petersen JW, Harboe ZB, et al. Antibody responses to pertussis toxin display different kinetics after clinical Bordetella pertussis infection than after vaccination with an acellular pertussis vaccine. J Med Microbiol. 2010;59(Pt 9):1029–1036. doi:10.1099/jmm.0.020826-0. 20508003

[11] Ryan M, Murphy G, Ryan E, et al. Distinct T-cell subtypes induced with whole cell and acellular pertussis vaccines in children. Immunology. 1998;93(1):1–10. doi:10.1046/j.1365-2567.1998.00401.x. 9536112 PMC1364099

[12] Salmaso S, Mastrantonio P, Wassilak SG, et al. Persistence of protection through 33 months of age provided by immunization in infancy with two three-component acellular pertussis vaccines. Stage II Working Group. Vaccine. 1998;16(13):1270–1275. doi:10.1016/S0264-410X(98)00040-1. 9682390

[13] Ausiello CM, Lande R, Urbani F, et al. Cell-mediated immunity and antibody responses to Bordetella pertussis antigens in children with a history of pertussis infection and in recipients of an acellular pertussis vaccine. J Infect Dis. 2000;181(6):1989–1995. doi:10.1086/315509. 10837180

[14] Liesenfeld O, Montoya JG, Kinney S, et al. Effect of testing for IgG avidity in the diagnosis of *Toxoplasma gondii* infection in pregnant women: experience in a US reference laboratory. J Infect Dis. 2001;183(8):1248–1253. doi:10.1086/319672. 11262207

[15] Almanzar G, Ottensmeier B, Liese J, et al. Assessment of IgG avidity against pertussis toxin and filamentous hemagglutinin via an adapted enzyme-linked immunosorbent assay (ELISA) using ammonium thiocyanate. J Immunol Methods. 2013;387(1-2):36–42. doi:10.1016/j.jim.2012.09.008. 23022630

[16] Denoel PA, Goldblatt D, de Vleeschauwer I, et al. Quality of the Haemophilus influenzae type b (Hib) antibody response induced by diphtheria-tetanus-acellular pertussis/Hib combination vaccines. Clin Vaccine Immunol. 2007;14(10):1362–1369. doi:10.1128/CVI.00154-07. 17699836 PMC2168112

[17] Ahmed R, Gray D. Immunological memory and protective immunity: understanding their relation. Science. 1996;272(5258):54–60. 8600537 10.1126/science.272.5258.54

[18] Goodnow CC, Vinuesa CG, Randall KL, et al. Control systems and decision making for antibody production. Nat Immunol. 2010;11(8):681–688. doi:10.1038/ni.1900. 20644574

[19] Barkoff AM, Grondahl-Yli-Hannuksela K, Vuononvirta J, et al. Differences in avidity of IgG antibodies to pertussis toxin after acellular pertussis booster vaccination and natural infection. Vaccine. 2012;30(48):6897–6902. doi:10.1016/j.vaccine.2012.09.003. 22981763

[20] McHeyzer-Williams MG, McLean MJ, Lalor PA, et al. Antigen-driven B cell differentiation in vivo. J Exp Med. 1993;178(1):295–307. doi:10.1084/jem.178.1.295. 8315385 PMC2191088

[21] Schallert N, Pihlgren M, Kovarik J, et al. Generation of adult-like antibody avidity profiles after early-life immunization with protein vaccines. Eur J Immunol. 2002;32(3):752–760. doi:10.1002/1521-4141(200203)32:3<752::AID-IMMU752>3.0.CO;2-5. 11870619

[22] Berek C, Berger A, Apel M. Maturation of the immune response in germinal centers. Cell. 1991;67(6):1121–1129. doi:10.1016/0092-8674(91)90289-B. 1760840

[23] Kepler TB, Perelson AS. Somatic hypermutation in B cells: an optimal control treatment. J Theor Biol. 1993;164(1):37–64. doi:10.1006/jtbi.1993.1139. 8264243

[24] French DL, Laskov R, Scharff MD. The role of somatic hypermutation in the generation of antibody diversity. Science. 1989;244(4909):1152–1157. 2658060 10.1126/science.2658060

[25] Goldblatt D, Vaz AR, Miller E. Antibody avidity as a surrogate marker of successful priming by Haemophilus influenzae type b conjugate vaccines following infant immunization. J Infect Dis. 1998;177(4):1112–1115. doi:10.1086/517407. 9534995

[26] Dorner T, Radbruch A. Antibodies and B cell memory in viral immunity. Immunity. 2007;27(3):384–392. doi:10.1016/j.immuni.2007.09.002. 17892847

[27] Prelog M, Almanzar G, Rieber N, et al. Differences of IgG antibody avidity after an acellular pertussis (aP) booster in adolescents after a whole cell (wcP) or aP primary vaccination. Vaccine. 2013;31(2):387–393. doi:10.1016/j.vaccine.2012.10.105. 23142306

[28] Eisen HN. Determination of antibody affinity for haptens and antigens by means of fluorescence quenching. Methods Med Res. 1964;10:115–121. 14284903

[29] Dimitrov JD, Lacroix-Desmazes S, Kaveri SV. Important parameters for evaluation of antibody avidity by immunosorbent assay. Anal Biochem. 2011;418(1):149–151. doi:10.1016/j.ab.2011.07.007. 21803020

[30] Pullen GR, Fitzgerald MG, Hosking CS. Antibody avidity determination by ELISA using thiocyanate elution. J Immunol Methods. 1986;86(1):83–87. doi:10.1016/0022-1759(86)90268-1. 3944471

[31] Hendrikx LH, Berbers GA, Veenhoven RH, et al. Igg responses after booster vaccination with different pertussis vaccines in Dutch children 4 years of age: effect of vaccine antigen content. Vaccine. 2009;27(47):6530–6536. doi:10.1016/j.vaccine.2009.08.052. 19729085

[32] Moriuchi T, Otsuka N, Hiramatsu Y, et al. A high seroprevalence of antibodies to pertussis toxin among Japanese adults: qualitative and quantitative analyses. PLoS One. 2017;12(7):e0181181. doi:10.1371/journal.pone.0181181. 28700751 PMC5507317

[33] Hovingh ES, Kuipers B, Bonacic Marinovic AA, et al. Detection of opsonizing antibodies directed against a recently circulating Bordetella pertussis strain in paired plasma samples from symptomatic and recovered pertussis patients. Sci Rep. 2018;8(1):12039. doi:10.1038/s41598-018-30558-8. 30104573 PMC6089961

[34] Pichichero M, Papa T, Blatter M, et al. Immune memory in children previously vaccinated with an experimental quadrivalent meningococcal polysaccharide diphtheria toxoid conjugate vaccine. Pediatr Infect Dis J. 2006;25(11):995–1000. doi:10.1097/01.inf.0000243215.46312.4a. 17072120

[35] Pichichero ME, Voloshen T, Zajac D, et al. Avidity maturation of antibody to Haemophilus influenzae type b (Hib) after immunization with diphtheria-tetanus-acellular pertussis-hib-hepatitis B combined vaccine in infants. J Infect Dis. 1999;180(4):1390–1393. doi:10.1086/314989. 10479180

[36] Dalby T, Seier-Petersen M, Kristiansen MP, et al. Problem solved: a modified enzyme-linked immunosorbent assay for detection of human antibodies to pertussis toxin eliminates false-positive results occurring at analysis of heat-treated sera. Diagn Microbiol Infect Dis. 2009;63(4):354–360. doi:10.1016/j.diagmicrobio.2008.12.004. 19249174

[37] He Q, Mertsola J, Himanen JP, et al. Evaluation of pooled and individual components of Bordetella pertussis as antigens in an enzyme immunoassay for diagnosis of pertussis. Eur J Clin Microbiol Infect Dis. 1993;12(9):690–695. doi:10.1007/BF02009381. 8243485

[38] Versteegen P, Valente Pinto M, Barkoff AM, et al. Responses to an acellular pertussis booster vaccination in children, adolescents, and young and older adults: A collaborative study in Finland, the Netherlands, and the United Kingdom. EBioMedicine. 2021;65:103247. 33647770 10.1016/j.ebiom.2021.103247 PMC7920834

[39] Reizenstein E, Hallander HO, Blackwelder WC, et al. Comparison of five calculation modes for antibody ELISA procedures using pertussis serology as a model. J Immunol Methods. 1995;183(2):279–290. doi:10.1016/0022-1759(95)00067-K. 7602150

[40] Raya BA, Bamberger E, Almog M, et al. Immunization of pregnant women against pertussis: the effect of timing on antibody avidity. Vaccine. 2015;33(16):1948–1952. doi:10.1016/j.vaccine.2015.02.059. 25744227

[41] Cabore RN, Maertens K, Dobly A, et al. Influence of maternal vaccination against diphtheria, tetanus, and pertussis on the avidity of infant antibody responses to a pertussis containing vaccine in Belgium. Virulence. 2017;8(7):1245–1254. doi:10.1080/21505594.2017.1296998. 28277900 PMC5711396

[42] Fumimoto R, Otsuka N, Sunagawa T, et al. Age-related differences in antibody avidities to pertussis toxin and filamentous hemagglutinin in a healthy Japanese population. Vaccine. 2019;37(18):2463–2469. doi:10.1016/j.vaccine.2019.03.055. 30930008

[43] Mosley YC, Radder JE, Berndt A, et al. Genome-wide association mapping of the antibody response to diphtheria, tetanus and acellular pertussis vaccine in mice. J Infect Dis. 2017;215(3):466–474. 28011915 10.1093/infdis/jiw587

[44] Ibrahim NM, El-Kady EM, Eissa SA, et al. Assessment of antibody concentration and avidity against Bordetella pertussis in a cohort of Egyptian individuals aged 1-18 years. J Adv Res. 2016;7(1):105–111. doi:10.1016/j.jare.2015.03.002. 26843976 PMC4703483

[45] Maertens K, Hoang THT, Cabore RN, et al. Avidity of maternal pertussis antibodies after vaccination during pregnancy. Vaccine. 2015;33(42):5489. doi:10.1016/j.vaccine.2015.05.075. 26116248

[46] Abu-Raya B, Giles ML, Kollmann TR, et al. Profiling avidity of antibodies elicited by vaccination using enzyme-linked immunosorbent assay-based elution - insights into a novel experimental and analytical approach. Vaccine. 2020;38(34):5389–5392. doi:10.1016/j.vaccine.2020.06.060. 32620372

[47] Barkoff AM, Knuutila A, Mertsola J, et al. Evaluation of anti-PT antibody response after pertussis vaccination and infection: The importance of both quantity and quality. Toxins (Basel). 2021;13(8):10. doi:10.3390/toxins13080508. PMC8402585 34437379

[48] Kneitz RH, Schubert J, Tollmann F, et al. A new method for determination of varicella-zoster virus immunoglobulin G avidity in serum and cerebrospinal fluid. BMC Infect Dis. 2004;4(33). doi:10.1186/1471-2334-4-33. PMC522815 15355548

[49] Bobic B, Klun I, Vujanic M, et al. Comparative evaluation of three commercial Toxoplasma-specific IgG antibody avidity tests and significance in different clinical settings. J Med Microbiol. 2009;58(Pt 3):358–364. doi:10.1099/jmm.0.006668-0. 19208887

[50] Marchant A, Pihlgren M, Goetghebuer T, et al. Predominant influence of environmental determinants on the persistence and avidity maturation of antibody responses to vaccines in infants. J Infect Dis. 2006;193(11):1598–1605. doi:10.1086/503775. 16652290

[51] Luijkx T, van Dijken H, van Els C, et al. Heterologous prime-boost strategy to overcome weak immunogenicity of two serosubtypes in hexavalent Neisseria meningitidis outer membrane vesicle vaccine. Vaccine. 2006;24(10):1569–1577. doi:10.1016/j.vaccine.2005.10.003. 16298029

[52] Luijkx TA, Brink v-v, van Dijken HH, et al. Hyperproliferation of B cells specific for a weakly immunogenic PorA in a meningococcal vaccine model. Clin Vaccine Immunol. 2008;15(10):1598–1605. doi:10.1128/CVI.00192-08. 18768670 PMC2565936

[53] Knuutila A, Dalby T, Barkoff AM, et al. Differences in epitope-specific antibodies to pertussis toxin after infection and acellular vaccinations. Clin Transl Immunology. 2020;9(8):e1161. doi:10.1002/cti2.1161. 32765879 PMC7396262

[54] Matos DC, Silva AM, Neves PC, et al. Pattern of functional antibody activity against Haemophilus influenzae type B (Hib) in infants immunized with diphtheria-tetanus-pertussis/Hib Brazilian combination vaccine. Braz J Med Biol Res. 2009;42(12):1242–1247. doi:10.1590/S0100-879X2009005000039. 19893995

[55] Marchant A, Kollmann TR. Understanding the ontogeny of the immune system to promote immune-mediated health for life. Front Immunol. 2015;6:77. 25755655 10.3389/fimmu.2015.00077 PMC4337332

[56] Prince HE, Lieberman JM, Cherry JD. Age-related differences in patterns of increased Bordetella pertussis antibodies. Clin Vaccine Immunol. 2012;19(4):545–550. doi:10.1128/CVI.05725-11. 22357646 PMC3318290

[57] Minh NNT, He Q, Ramalho A, et al. Acellular vaccines containing reduced quantities of pertussis antigens as a booster in adolescents. Pediatrics. 1999;104(6):e70. doi:10.1542/peds.104.6.e70. 10586004

[58] Knuutila A, Versteegen P, Barkoff AM, et al. Pertussis toxin neutralizing antibody response after an acellular booster vaccination in Dutch and Finnish participants of different age groups. Emerg Microbes Infect. 2022;11(1):956–963. doi:10.1080/22221751.2022.2053364. 35286231 PMC8973383

[59] Versteegen P, Barkoff AM, Valente M, et al. Memory B cell activation induced by pertussis booster vaccination in four age groups of three countries. Front Immunol. 2022;13:864674. doi:10.3389/fimmu.2022.864674. 35677044 PMC9168128

[60] Guiso N, Berbers GA, Fry NK, et al. What to do and what not to do in serological diagnosis of pertussis: recommendations from EU reference laboratories. Eur J Clin Microbiol Infect Dis. 2011;30(3):307–312. doi:10.1007/s10096-010-1104-y. 21069406 PMC3034915

